# Biomarkers in the Management of Complement-Mediated Kidney Diseases in the Era of Complement Therapeutics

**DOI:** 10.2215/CJN.0000000967

**Published:** 2025-11-25

**Authors:** Dana V. Rizk, Bradley P. Dixon, Melvin Chan, H. Terence Cook, Ashley Frazer-Abel, Sydney C.W. Tang

**Affiliations:** 1 Division of Nephrology, Department of Medicine, University of Alabama at Birmingham, Birmingham, Alabama; 2 Department of Pediatrics, University of Colorado School of Medicine, Aurora, Colorado; 3 Department of Immunology and Inflammation, Imperial College London, London, United Kingdom; 4 Division of Rheumatology, Department of Medicine, University of Colorado Anschutz Medical Campus, Aurora, Colorado; 5 Division of Nephrology, Department of Medicine, School of Clinical Medicine, The University of Hong Kong, Queen Mary Hospital, Hong Kong, China

**Keywords:** chronic GN, CKD, complement, histopathology, immunohistochemistry, glomerular diseases, biomarkers

## Abstract

Pharmacologic complement inhibition offers a promising therapeutic strategy for several complement-mediated kidney diseases. Yet, at present, nephrologists must rely on an incomplete toolkit of histopathologic and circulating biomarkers to assess complement activity in complement-mediated kidney diseases. Our clinical capacity to characterize and monitor pathologically dysregulated complement for disease prognosis, to inform patient selection, and to evaluate therapeutic efficacy severely lags behind the growing number of complement inhibitors under development. Reliable, sensitive complement biomarkers that are suitable for clinical assessment are needed for the timely and optimal implementation of existing and upcoming therapeutics. Despite this urgent need and growing research efforts, the repertoire of clinically available complement biomarker assays has proven refractory to expansion. This is, in part, due to a myriad of practical challenges limiting the information reliably interpreted from existing complement biomarkers and hindering the translation of novel biomarkers from research settings into the clinical pathology laboratory. In this article, the authors review commonly evaluated complement biomarkers within the context of an evolving therapeutic landscape, as well as the practical challenges related to their effective application. Noteworthy, emerging biomarkers are also discussed, along with the challenges in translating robust markers of complement activity from research settings into clinical practice.

## Introduction

**Figure 1:**
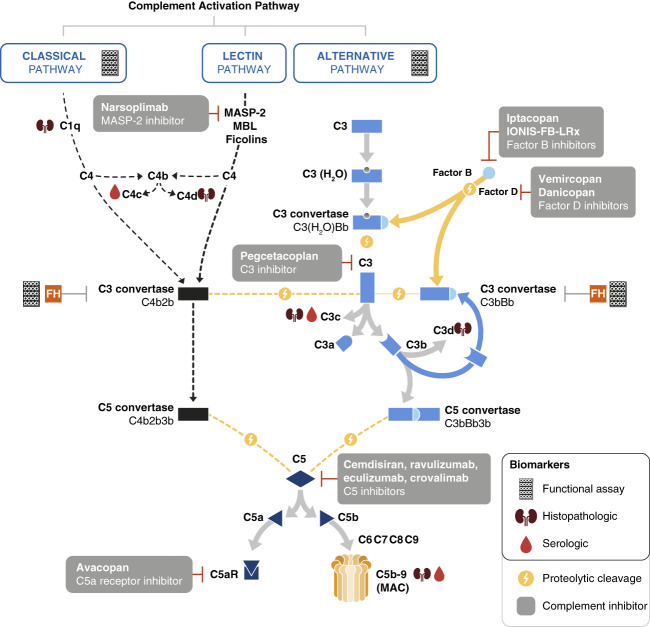
**Routinely used complement biomarkers and therapeutic complement inhibitors.** Avacopan is approved as an adjunctive treatment of ANCA-associated vasculitis in combination with standard therapy including glucocorticoids; crovalimab is approved for the treatment of paroxysmal nocturnal hemoglobinuria; danicopan is approved as an add-on to ravulizumab or eculizumab for the treatment of extravascular hemolysis in patients with paroxysmal nocturnal hemoglobinuria; eculizumab is approved for the treatment of aHUS, generalized myasthenia gravis, neuromyelitis optica spectrum disorder, and paroxysmal nocturnal hemoglobinuria; iptacopan is approved for the treatment of paroxysmal nocturnal hemoglobinuria, IgA nephropathy, and C3G; pegcetacoplan is approved for the treatment of paroxysmal nocturnal hemoglobinuria, C3G, and primary IC-MPGN; ravulizumab is approved for the treatment of aHUS, generalized myasthenia gravis, neuromyelitis optica spectrum disorder, and paroxysmal nocturnal hemoglobinuria. Cemdisiran, narsoplimab, IONIS-FB-LRx, and vemircopan are not currently FDA approved. Adapted and reprinted under the CC BY 4.0 license from ref. 57. https://creativecommons.org/licenses/by/4.0/. aHUS, atypical hemolytic uremic syndrome; C3G, C3 glomerulopathy; FDA, US Food and Drug Administration; FH, factor H; IC-MPGN, immune-complex membranoproliferative GN; MAC, membrane attack complex; MASP-2, mannose-associated serine protease 2; MBL, mannose-binding lectin.

The complement system is a major effector mechanism of the innate immune response. Three initiation pathways—the classical, lectin, and alternative pathways (APs)—converge at the activation of complement protein C3 and subsequently activate the terminal pathway, leading to the formation of the membrane attack complex (C5b-9, also known as the terminal complement complex) and proinflammatory anaphylatoxins, C3a and C5a. Together, these complement pathways elicit efficient and tightly regulated inflammatory and cytolytic responses against pathogens, damaged tissue, and other surfaces identified as “nonself” (Figure 1).^1^

Overactivation of the complement system can cause, or contribute to, glomerular injury.^2,3^ Complement-mediated kidney diseases (CMKDs) are a spectrum of rare disorders in which complement plays a key role in pathogenesis.^2,4^ The role of complement in CMKD pathophysiology varies greatly by disease and disease stage and has been reviewed extensively elsewhere.^2,4–7^ Prototypical CMKDs include C3 glomerulopathy (C3G), idiopathic immune-complex membranoproliferative GN (IC-MPGN), and atypical hemolytic uremic syndrome (aHUS). Complement overactivation also plays a role in IgA nephropathy, IgA-associated vasculitis with nephritis, ANCA-associated kidney vasculitis (AAV), membranous nephropathy (MN), and lupus nephritis (LN) and may contribute to kidney transplant rejection.^2,8^

For many CMKDs, a persisting lack of disease-modifying treatments has meant a poor prognosis, with patients failing to achieve disease control, experiencing disease progression, and disease recurrence post-transplant.^9^ However, pharmacologic complement inhibition offers a promising therapeutic strategy.^2^ Terminal pathway blockade with eculizumab, an anti-C5 antibody that blocks cleavage of C5 into C5a and C5b, has transformed the outlook for patients with aHUS.^10^ The terminal pathway-targeted medications ravulizumab (anti-C5), which was shown to reduce proteinuria and a trend toward stabilization of eGFR in patients with IgA nephropathy,^11^ and avacopan (anti-C5aR), have also received regulatory approval for the treatment of aHUS and AAV, respectively.^12,13^ More recently, the AP inhibitor iptacopan received US Food and Drug Administration (FDA) approval for IgA nephropathy and C3G.^14,15^ Furthermore, pegcetacoplan, a C3 and C3b inhibitor, has also received recent FDA approval for the treatment of C3G and primary IC-MPGN.^16^ These inhibitors, plus others, are under clinical investigation across multiple CMKDs (Figure 1).^17–20^

In CMKDs, pathologic complement components and activation fragments can localize within glomerular compartments and be detected in patients' serum, plasma, and urine, providing insights into pathway(s) and level of complement activation (Figure 1).^4,21^ Yet, our clinical capacity to characterize and monitor dysregulated aspects of the complement system continues to lag behind the growing number of complement inhibitors under development.^2,5,21^ The identification and validation of biomarkers to inform patient selection, prognosis, and responses to complement inhibitor treatment is an urgent research priority, as highlighted recently in the Kidney Disease: Improving Global Outcomes 2025 Clinical Practice Guideline for the Management of IgA Nephropathy and IgA Vasculitis.^22^

In this article, we review current knowledge of complement biomarkers, including their practical strengths and limitations, and discuss the challenges in translating robust markers of complement activity from research settings into clinical practice.

## Histopathologic Complement Biomarkers

There are several histopathologic stains for complement components that offer reliable insights into activation in patients with glomerular disease.^23,24^ Histopathologic complement biomarkers that are routinely assessed, as well as several that have shown promise in research settings, are summarized in Table 1.

**Table 1:** Histopathologic complement biomarkers in complement-mediated kidney diseases

Biomarker Category	Biomarker/Assay	Diagnostic/Prognostic	Utility as a Complement Biomarker
Routinely assessed in CMKDs	C3 (polyvalent)	Diagnostic	• Detects multiple fragments of C3 that are produced during the complement cascade^36^
	C3 (C3c)	Diagnostic	• Marker of recent or ongoing C3 activation^25^ • In the absence of classical and lectin pathway activation markers, may establish AP activation^23^ • C3 and Ig costaining can provide insight into complement overactivation and immune complex localization to inform diagnosis of various CMKDs^5,36^ • Utility limited by rapid dissociation from binding sites^25^
	C3 (C3d)	Diagnostic	• Marker of C3 activation in the classical and APs^26^ • Remains attached to binding sites for long periods, leaving persistent evidence of complement activation^26^ • Cannot be used as an indicator of current complement activity^25^
	C1q	Diagnostic	• Marker of classical pathway activation^27^ • Presence or absence helps to distinguish various CMKDs^2,27^
	C4d	Diagnostic	• Marker of classical pathway activation (with C1q) and lectin pathway activation (in the absence of C1q)^28,35^ • Utility limited due to weaker staining intensity in formalin-fixed tissue and potential for detection by immunofluorescence in normal glomeruli^30^
		Prognostic	• Associated with worse prognosis for patients with IgA nephropathy in native kidneys^29^
	C5b-9	Diagnostic	• Marker of terminal pathway activation^32^ • Positive C5b-9 staining is observed in patients with LN^32^
		Prognostic	• Glomerular C5b-9 deposits may be associated with poor survival in some patients with MN^31^ and C3G^63^ and correlates with disease progression in patients with C3G and IgA nephropathy^63,64^
Currently under investigation	MBL	Diagnostic	• Glomerular MBL deposition can be detected in up to 50% of patients with MN^4^
		Prognostic	• Glomerular MBL deposition has also been observed in patients with IgA nephropathy in whom it may indicate poor prognosis^51^
	Factor B	Diagnostic	• Immunofluorescence staining of factor B can indicate the role of the AP in patients with MN^2,72^
	FHR5	Diagnostic	• Positive FHR5 staining is associated with active disease in IgA nephropathy^101,133^
		Prognostic	• Positive FHR5 staining is also associated with disease severity in C3G and IgA nephropathy^101,134^

AP, alternative pathway; C3G, C3 glomerulopathy; CMKD, complement-mediated kidney disease; FHR5, factor H–related protein 5; LN, lupus nephritis; MBL, mannan-binding lectin; MN, membranous nephropathy.

### In Clinical Practice

**Figure 2:**
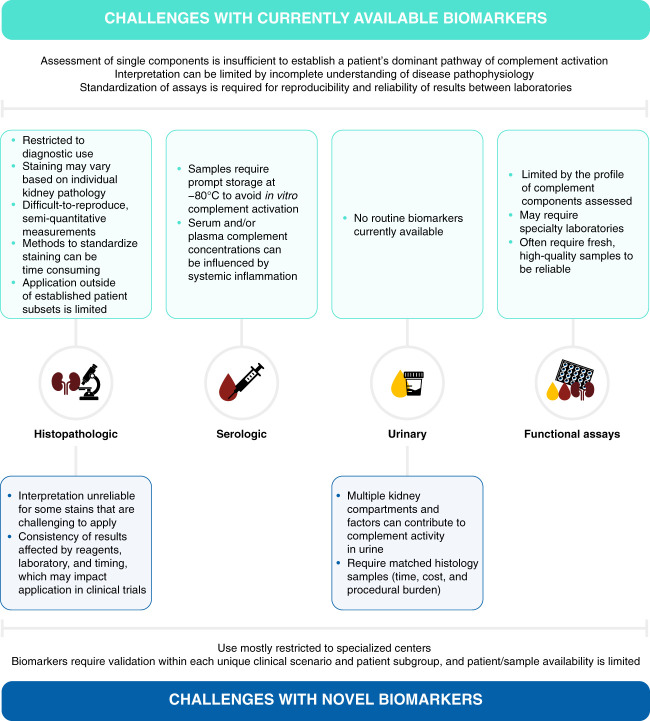
Practical challenges in the clinical application of complement biomarkers.

Currently, analysis of kidney biopsies using immunofluorescence or immunohistochemistry (IHC), which have been shown to be comparable methods for the detection of Ig, is the gold standard for the diagnosis of glomerular diseases, and the predominant method recommended to establish complement involvement. Routine diagnostic evaluation of complement is often limited to immunofluorescence staining of C3 and C1q, which can provide insights into lectin and AP activation versus classical pathway activation (Table 1).^4,23,25–36^ Although histologic biomarkers are considered robust and reliable, there are considerations and limitations to their use. For example, high background noise or off-target binding can reduce reliability and reproducibility,^30,37–39^ and the interpretation of tissue staining can be complicated by nonspecific binding,^40^ a lack of a uniform definition for staining positivity, and the absence of immunofluorescence criteria that have been validated in a well-defined disease cohort (Figure 2).^41^

#### Complement C1q

Immunofluorescence analysis of glomerular deposition of C1q is part of routine kidney biopsy evaluation in glomerular disease.^23^ C1q deposits are a distinctive finding in patients with LN, underpinning the significance of C1q in the inflammatory mechanisms associated with this condition.^42^ Histopathologic C1q staining can also help clinicians establish or rule out classical pathway activation in patients with C3G,^43^ MN,^44^ AAV,^45,46^ or IgA nephropathy.^23^ Similarly, the presence or absence of C1q staining can help to distinguish between IC-MPGN (present at varying intensities) and C3G (typically absent).^2^ In patients with IgA nephropathy, C1q deposition is rare and is associated with more severe clinicopathologic features and worse prognosis.^47,48^

#### Complement C4d

For biomarkers of lectin pathway overactivation, assessment of C4d (which indicates lectin pathway activity in the absence of positive C1q staining) is widely available and currently included in IHC staining panels for kidney allograft biopsies as a biomarker of antibody-mediated rejection.^28,30,49^ C4d activation in subsets of patients with IgA nephropathy (up to one third of patients) or MN (up to 100% of patients) is well documented, and C4d deposition has been frequently associated with increased proteinuria and worse prognosis in studies of glomerular diseases.^29,35,37,50,51^ For example, in patients with IgA nephropathy, C4d staining has been identified as a marker of disease progression and a potential independent risk factor for late-stage kidney disease.^29^ Nonetheless, routine staining of C4d has not yet expanded to native kidney biopsies,^49^ possibly due to the weaker staining intensity of C4d IHC in formalin-fixed tissue compared with immunofluorescence on frozen sections, a lack of uniform definition of C4d positivity in clinical practice, or the potential for C4d detection in normal glomeruli by immunofluorescence.^30,38^ In addition, C4d covalently binds to cell surfaces, remaining at the site of complement activation long after activity has ceased.^49^ This provides valuable insights into sites of antibody-mediated injury, particularly in the transplant setting,^49^ but may have disadvantages regarding the value of C4d within treatment monitoring.

#### Complement C3

The presence of C3 deposits may establish AP overactivation, a driver of pathology in C3G and AAV, and C3 staining is the cornerstone of C3G diagnosis.^2,24,52^ C3 is a large and structurally complex complement protein which undergoes fragmentation during activation; thus, in practice, there are different stains that can be used including a polyvalent C3 stain and a C3c stain. In most pathology laboratories, C3 is detected using an antibody to C3c, a biologically inactive C3 breakdown product.^25,40^ Owing to the rapid dissociation of C3c from binding sites, glomerular C3c can act as a biomarker for recent or ongoing C3 overactivation.^25,40^ A study found that biopsies from some patients with C3G may stain negative for C3c; therefore, polyvalent C3 staining may be a more robust method. Costaining for C3 and Ig can provide initial insight into complement overactivation and the localization of immune complexes within distinct glomerular compartments, thereby informing or ruling out a diagnosis of immune complex-mediated disease. For example, immunofluorescence staining of C3 and Ig is essential to distinguish between C3G and IC-MPGN, which share a pattern of membranoproliferative GN injury by light microscopy on kidney biopsy.^52,53^ Specifically, the intensity of C3 immunofluorescence staining is an important differentiation: dominant mesangial and capillary wall C3 staining is observed in C3G (at least two-fold higher than for any other immune reactant), whereas in IC-MPGN, codeposition of C3 with Ig is seen.^52–54^ Importantly, there is the possibility of “masked” monoclonal Ig deposits in IC-MPGN, which are deposits undetectable by standard immunofluorescence of fresh-frozen sections that are only revealed by immunofluorescence of protease-digested paraffin sections.^55^

Biopsies from patients with primary MN typically exhibit strong IgG and bright capillary wall C3 staining by immunofluorescence,^23,44^ and C3 glomerular deposition is often seen in IgA nephropathy.^5^ Studies in IgA nephropathy have also linked C3 deposition and immunofluorescence staining intensity with disease severity and progression risk,^28,56^ prompting debate as to whether C3 immunofluorescence should become a standardized prognostic evaluation in addition to Oxford Classification MEST-C (mesangial [M] and endocapillary [E] hypercellularity, segmental sclerosis [S], interstitial fibrosis/tubular atrophy [T], and crescents [C]) criteria.^57^ However, C3 has been observed in areas of hyalinosis in arterioles, which is frequently associated with glomerulosclerosis, in hypertension-associated kidney disease, and in trace amounts in the glomeruli, attributable to nonspecific trapping in transplant glomerulopathy.^58,59^ Thus, care should be taken to confirm the underlying mechanism responsible for C3 deposition.

Notably, owing to weak signal in formalin-fixed and paraffin-embedded specimens, C3 staining can result in high interobserver variability.^39,41^ This has implications for the utility of glomerular C3 staining intensity as a diagnostic criterion and its potential future use in guiding patient eligibility for therapeutic complement inhibition. Methods to standardize assessment of histopathologic staining, such as image analyses, are currently limited by their time-consuming nature—developing objective quantitation methods by leveraging recent advances in artificial intelligence technology within image analysis may help to overcome this.

Glomerular C3d is an additional biomarker of C3 activation, assessed by immunofluorescence or IHC. Unlike C3c, which rapidly dissociates, C3d covalently binds and remains attached to cell surfaces, persisting in glomerular tissue after C3 activation has ceased.^25,26^ In AAV, glomerular C3d may provide prognostic insights and has been associated with poor kidney survival and cellular crescent formation.^60,61^ In IgA nephropathy, C3d deposition may act as an indicator of chronic, inactive phases of disease, during which C3c deposition may be decreased. C3d IHC has also been suggested as a complementary tool to detect C3 deposits in the diagnosis of C3G.^62^

#### Complement C5b-9

C5b-9, a critical effector of the terminal complement pathway, can be overproduced in CMKDs due to terminal pathway overactivation. Histopathologic stains for C5b-9 are available and can reliably detect C5b-9 production, for example, in patients with LN.^32^ C5b-9 is also detected in patients with MN and has been associated with poor kidney survival.^31^ In addition, in patients with C3G and IgA nephropathy, the amount of C5b-9 deposition correlates with disease prognosis.^63,64^ However, the utility of glomerular C5b-9 as a biomarker remains limited due to variable rates of clearance and a long *t*_1/2_, which results in persistent staining (months to years)^32^ that can be comparable in patients with active or chronic disease states.^32^ Thus, it is unlikely that glomerular C5b-9 staining will inform management approaches in patients with CMKDs in the future.

#### Inflammatory versus Fibrotic Phenotypes

In patients with CMKDs, it may be important to differentiate between inflammatory and fibrotic phenotypes when making treatment decisions. Patients' disease can present along a spectrum ranging from active glomerular inflammation to chronic/irreversible damage, marked by fibrosis.^65^ Activation of the complement pathway in CMKDs is multiform, including the deposition of complement degradation products in the glomeruli and glomerular immune complexes.^5^ This activation can lead to the production of C3a and C5a, which recruit inflammatory cells to the site of inflammation, thus contributing to the development of interstitial fibrosis.^5^ In IgA nephropathy, C3 deposition and increased plasma C3 fragments correlate with more severe disease, thus potentially supporting the role of complement in driving inflammation.^66–68^ However, the relationship between complement proteins and inflammation in patients with CMKDs is yet to be fully elucidated. There is some evidence that complement activation may contribute to the clearance of antigen-antibody complexes in tissues, therefore preventing injury through inflammation.^69,70^ In addition, complement activation within the tubulointerstitial compartment has also increasingly been recognized as relevant to fibrosis and disease progression in CKD.^71^

### In Research

Several histopathologic complement biomarkers used in research settings have shown potential for the diagnosis, prognostication, and monitoring of CMKDs (Table 1). For instance, studies have detected glomerular mannose-binding lectin deposition in approximately 50% of patients with MN and in approximately 25% of patients with IgA nephropathy, in whom it may be associated with adverse kidney outcomes.^4,51^ Notable research-only histopathologic biomarkers of the AP include factor B, which can indicate AP involvement in patients with MN,^2,72^ and factor H (FH)–related protein 5, which may be a marker of disease severity in patients with IgA nephropathy.

However, the application of many histopathologic stains for complement biomarkers remains restricted to specialized research centers. This is in part due to the rare nature of most CMKDs such that evidence on candidate complement biomarkers has largely been generated from observational, single-center studies conducted at specialized facilities. Furthermore, barriers to the successful validation of novel biomarkers (Figure 2), such as limited patient/sample availability, remain amplified in rare diseases.

## Noninvasive Complement Biomarkers

Kidney biopsies provide limited insights on the contribution of complement and offer only a single snapshot in time. Although repeat biopsies can assess disease course, undertaking multiple biopsies is not feasible in clinical practice due to the invasive nature of the procedure. Thus, noninvasive complement biomarkers could facilitate patient selection and treatment monitoring for the optimal implementation of novel complement inhibitors.

### In Routine Clinical Practice

At present, clinicians have a basic repertoire of serologic complement biomarkers and functional assays at their disposal (Table 2).^2^ Moreover, urinary biomarkers are not used in routine clinical practice.^21^

**Table 2:** Noninvasive complement biomarkers in complement-mediated kidney diseases

Biomarker Category	Biomarker	Diagnostic/Prognostic	Significance and Clinical Scenario
Routinely assessed in CMKDs	Circulating C3 (C3c)/C4 (C4c)	Diagnostic	• Marker of C3 activation^21^ • C3c and C4c cannot distinguish intact C3 or C4, and fragmentation may occur with poor sample storage and increased sample age^21^
		Prognostic	• Included in common disease activity scores for LN^135^; low circulating levels are associated with disease activity and responses to belimumab^136–138^ • C3 levels correlate with histologic activity score in C3G^108^ • Low serum C3 is associated with increased risk of kidney failure for IgA nephropathy and LN^77,78^
	CH50/liposomal CH50 functional assay	Diagnostic	• Evaluates classical pathway activation^21^ • Variations on the original hemolytic CH50 assay include liposome-based and ELISA-based CH50 assays, which vary in sensitivity^2,21,85^
	AH50/AP50 functional assay	Diagnostic	• Evaluates AP activation^21^ • In patients with AP overactivation (such as patients with aHUS or C3G), low AH50 activity can reflect consumption of AP components or indicate a deficiency in factor B, factor D, FH, or properdin^84,85^
	FH functional assay	Diagnostic	• Evaluates FH activity; FH deficiency can result in AP overactivation^85,86^ • Can aid in establishing AP overactivation, particularly when AP50 activity is low, but should be interpreted in the context of other complement assays^85^ • May be used in the diagnosis of aHUS and C3G^85^
	NeFs	Diagnostic	• NeFs are a group of autoantibodies which are a common driver of C3G^94–96^ • May be used to aid diagnosis; however, interpreting these tests remains challenging^94^
In research	Serum IgA:C3 ratio	Diagnostic	• Serum IgA:C3 ratio has been widely studied in IgA nephropathy and shows potential as a diagnostic tool^75,98^
		Prognostic	• Serum IgA:C3 ratio has also been independently associated with progression to kidney failure in cohort studies of patients with IgA nephropathy^99,100^
	Serologic and urinary C3a	Diagnostic	• Increased circulating and urinary C3a reflects complement activation in CMKDs including MN,^106,123^ and C3a shows potential as a biomarker of complement activity,^5^ including in patients with AAV^4,115^
	Urinary C3d	Diagnostic	• In patients with LN, urinary C3d may be a marker of active disease^118,139^
		Prognostic	• Urinary C3d may also be used to monitor disease activity, assess treatment response, and predict disease flare in patients with LN^115^
	Serologic MASP-1	Diagnostic	• Plasma levels of MASP-1 have been shown to be increased in, and may aid in diagnosis of, patients with IgA nephropathy and patients with LN^101,102^
	Serologic Bb	Prognostic	• Increasing serologic Bb levels are associated with worse outcomes in patients with IgA nephropathy, including a higher likelihood of kidney failure^104^
	Urinary Bb	Prognostic	• Urinary Bb correlates with disease activity in AAV and levels appear to normalize in patients in remission^115,120^
	Serologic Ba	Diagnostic	• Elevated levels of plasma Ba have been observed in patients with IgA nephropathy compared with control patients^103^
	Urinary Ba	Diagnostic	• Urinary Ba was shown to be significantly higher in patients with C3G compared with healthy controls^115^
		Prognostic	• Urinary Ba may also serve as a biomarker of both disease activity and treatment response in patients with C3G^115^
	Serologic C5	Prognostic	• Serologic C5 may be useful to monitor relapse risk in patients with aHUS^2^ and correlates with histologic activity score in C3G^108^
	Urinary C5a	Diagnostic	• Increased urinary C5a reflects complement activation in multiple CMKDs, including MN and LN^122,123^
		Prognostic	• Urinary C5a also shows potential as a biomarker to monitor response to complement blockade in aHUS^124^
	Serologic sC5b-9	Diagnostic	• High levels of circulating sC5b-9 correlated with histologic activity score in C3G^108^ and have been reported in a study of patients with LN^140^
		Prognostic	• High levels of sC5b-9 have been observed in patients with active AAV compared with disease remission^106^ • A serum-induced C5b-9 endothelial deposition test in patients with aHUS has shown an ability to monitor patients' disease activity^105^
	Urinary sC5b-9	Diagnostic	• Urinary sC5b-9 levels appear to correlate with plasma levels of sC5b-9, which in turn correlate with histologic activity score in C3G^108^ and with complement overactivation in aHUS,^124^ AAV,^115^ LN,^115^ and MN^4,125^

AAV, ANCA-associated kidney vasculitis; AH50, alternative pathway hemolytic activity 50%; aHUS, atypical hemolytic uremic syndrome; AP, alternative pathway; C3G, C3 glomerulopathy; CH50, complement hemolytic activity 50%; CMKD, complement-mediated kidney disease; FH, factor H; LN, lupus nephritis; MASP-1, mannose-associated serine protease-1; MN, membranous nephropathy; NeF, nephritic factor; sC5b-9, soluble C5b-9.

#### Serologic Biomarkers

Circulating C3 and C4 are currently the only noninvasive complement biomarkers routinely assessed in patients with glomerular disease and are detected through antibodies targeting C3c or C4c in a similar approach to histologic assessment of C3 and C4. Typically, low circulating C3 or C4 indicates increased complement consumption as a result of pathway overactivation. However, glomerular complement activation is not always reflected by systemic hypocomplementemia—many patients adequately compensate for consumption with robust complement production.^4,73^ Up to 81% of patients with IgA nephropathy^74,75^ and up to 41% of patients with C3G^73^ have normal serum or plasma C3 levels. Hypocomplementemia may also be masked in patients with inflammation due to an increase in hepatic production of complement proteins.^76^ However, low serum C3 has been associated with an increased risk of progression to kidney failure in patients with IgA nephropathy and LN.^77,78^ Importantly, the antibodies used for these biomarkers react with C3c or C4c, as well as intact C3 or C4 (respectively), and do not generally distinguish intact components from breakdown products.^21^

#### Functional Complement Assays

Several functional assays of complement activity are available and have been used in clinical trials of investigational complement inhibitors.^20,79,80^ The original complement hemolytic activity 50% complement function assay, for investigation of classical pathway activity, is still available and has high sensitivity when performed by a qualified laboratory.^21^ Other methods such as ELISA-based or liposomal-based classical pathway function testing may also be used.^21^ Currently, many US hospital laboratories use high-throughput liposomal assays to assess classical pathway function, which may not have sufficient sensitivity for patient monitoring.^21,81^

AP hemolytic activity 50% assays (AH50, also known as the AP50 assay) are currently available to qualitatively determine AP activity, although ELISA-based methods are becoming more common.^21^ The Wieslab Complement System AP assay is used in research settings to monitor complement blockade in patients with aHUS receiving eculizumab^82^ and in clinical studies of iptacopan.^19,80,83^ In these clinical trials, low AH50/AP activity reflected therapeutic complement inhibition.^80,82,83^ In untreated patients with AP overaction (such as patients with aHUS or C3G), low AH50/AP activity may also reflect activation-related consumption of AP components or indicate a deficiency in factor B, factor D, FH, or properdin.^84,85^ Hemolytic functional assays for FH, a regulator of the AP C3 convertase, are also available and can aid in establishing AP overaction when AH50/AP activity is low.^85,86^

Caution should be taken when interpreting results as these assays require technical expertise and use of high-quality samples.^21^ Complement is the heat-labile fraction of the immune system, so samples need to be assayed immediately or frozen at −80°C within 2 hours of venipuncture.^87,88^

#### Monitoring Complement Inhibitors

Functional complement and biomarker assays can also play an important role in monitoring the treatment response of patients under complement blockade therapy (Table 2). For example, in patients with aHUS, following C5 blockade, there were reductions in complement hemolytic activity 50%, AP50, C3d, and soluble C5b-9 (sC5b-9) levels.^89^ In addition, factor B inhibition in patients with paroxysmal nocturnal hemoglobinuria, C3G, and IgA nephropathy decreased plasma Bb, Wieslab activity, and sC5b-9 levels, demonstrating effective AP blockade.^90^ Interpreting complement biomarkers to assess treatment efficacy is not always clear. For example, avacopan is a C5a receptor inhibitor approved for the treatment of AAV, but despite high remission rates, there is a lack of identified complement biomarkers for monitoring of treatment efficacy.^13,91^ Studies have shown an increase in serum C5a as a result of avacopan treatment in patients with AAV and have used CD11b as a marker of C5a receptor blockade.^91,92^ In addition, treatment with the C3 inhibitor pegcetacoplan does not show substantive inhibition in some complement functional assays, particularly those of the classical pathway, and serum C3 levels have been shown to increase despite good evidence for complement inhibition in patients.^79,93^ This could be due to the mode of action and an increased circulating pool of C3 bound to the inhibitor, thus reducing C3 activation. Nevertheless, these assays may play a valuable role in developing individualized treatment regimens through monitoring complement activity.

#### Nephritic Factors

Nephritic factors (NeFs) are a group of autoantibodies which target neoepitopes generated by the C3 (C3NeF) and C5 (C5NeF) convertases of the complement system.^94^ In C3G, NeFs are a common driver of disease, with C3NeFs occurring in ≥50% of cases and C5NeFs in approximately 50%.^95,96^ Furthermore, autoantibodies against the classical/lectin pathway C3 convertase (C4NeF) have also been identified in patients with C3G (5%–15%), albeit at lower rates.^97^ Owing to this, recent expert opinions have recommended using autoantibody assays against NeFs to aid diagnosis. There are several assays available to characterize the mechanistic action of NeFs; however, interpreting these tests, including their relationship to *in vivo* complement biomarkers, and differentiating C3NeFs from C5NeFs remains challenging.^94,96^

### In the Research Laboratory

#### Circulating Markers of Complement Activation

Several serologic biomarkers have shown promise for the analysis of complement in patients with CMKDs (Table 2), including serum IgA:C3 ratio for the diagnosis and monitoring of IgA nephropathy,^75,98–100^ C3a as a biomarker of C3 activity in patients with AAV, and elevated mannose-associated serine protease-1 to aid diagnosis in patients with IgA nephropathy or LN.^101,102^ In addition, circulating AP components Bb and Ba have been evaluated as biomarkers for diagnosis and disease monitoring in IgA nephropathy.^103,104^ A serum-induced *ex vivo* sC5b-9 endothelial deposition test has been evaluated in patients with aHUS and has shown an ability to monitor patients' disease activity.^105^ In addition, C5a and sC5b-9 have shown potential for monitoring relapse risk in aHUS and disease activity in AAV, respectively,^2,106,107^ as well as correlating with histologic activity score in C3G.^108^

Assessment of circulating complement components has technical considerations; complement activation may occur or be amplified *ex vivo* because complement, as previously mentioned, is not thermally stable.^21,87,88^ Therefore, appropriate sample handling is required (*i.e*., storage at −80°C within 2 hours), which can be challenging in some settings.^21,88^ In addition, sample type is critical for the accurate measurement of biomarkers of complement activation; specifically, EDTA plasma is the required sample type as the EDTA chelates the Ca^2+^ required by the classical and lectin pathways and the Mg^2+^ required for the AP.^109,110^ Serum samples, or other sample types, can allow *ex vivo* activation of the complement pathways and may lead to artificially high measurements of markers such as C3a and sC5b-9.^88,110,111^ The practical limitations of methods used to investigate noninvasive complement biomarkers have been discussed comprehensively in recent reviews.^21,111,112^ In addition, complement activation products may accumulate in circulation as kidney function declines.^113^ Furthermore, vascular complement proteins may become concentrated in kidney capillaries because of the filtration process, and these high concentrations may lead to complement-mediated kidney damage.^114^

#### Urinary Biomarkers

The assessment of urinary complement biomarkers may more precisely reflect intrarenal inflammation than traditional circulating biomarkers that can reflect chronic systemic inflammation.^115–117^ For example, circulating C3, a biomarker of inflammation occurring due to generalized immune activation, has shown low sensitivity at predicting kidney flares in LN, as demonstrated by a small longitudinal study comprising 71 patients with LN followed for approximately 3 years. Of interest, urinary C3d levels appear elevated in active disease states in LN,^118,119^ more accurately differentiating active from inactive LN than circulating C3, C4, C4d, or C5b-9.

In addition, several complement components have been evaluated as promising urinary biomarkers in research settings (Table 2). In patients with AAV, urinary Bb was found to be higher in active disease states, with levels appearing to normalize in patients in remission.^115,120^ Similarly, in C3G, urinary Ba has been shown to be significantly higher in patients compared with healthy controls, as well as higher in those with AKI compared with those with recovered kidney function. Thus, urinary Ba may be a biomarker of both disease activity and treatment response.^115,121^ Increased urinary C5a may be a marker of complement activation in multiple CMKDs including MN and LN.^122,123^ Urinary sC5b-9 levels appear to correlate with plasma sC5b-9 levels, which in turn correlate with histologic activity score in C3G,^108^ with complement overactivation in aHUS,^124^ AAV,^115^ LN,^115^ and MN,^4,125^ and with treatment response to complement blockade in aHUS, AAV, and LN. Recent research has also identified urinary C5b-9 as the best measure of histologic LN activity, over C5a and Ba.^126^ However, additional research is required before translation into clinical practice, as it has also been shown that urinary sC5b-9 is not a consistent biomarker of complement-mediated inflammation and complement deposition.^127^ The high mol wt of sC5b-9 means that it is not filtered by the glomeruli even with heavy proteinuria.^127^ The source of urinary sC5b-9 remains to be elucidated; however, it may occur due to intrarenal complement activation that is not characterized by complement glomerular deposition.^127^ Therefore, caution should be taken when interpreting urinary sC5b-9. In general, the pathologic significance of increased urinary complement components and activation fragments is often unclear, and matched histology samples remain important to the interpretation of urinary complement levels.^128^ It is also important to note that complement biomarkers present in both serum and urine may be a result of systemic inflammation rather than kidney-specific inflammation, which may make interpretation challenging.

## Conclusion

The timely and optimal implementation of complement inhibitors within CMKDs is linked to the availability of complement biomarkers that are robust enough to inform clinical decision-making.^2,22^ Complement biomarker analysis may also improve market access to complement inhibitors by providing insights into the patient populations potentially eligible for reimbursement. With the appropriate evidence base, biomarkers could be leveraged through development of histologic “complement panels” for use at diagnosis, or through incorporation of complement biomarker analyses into prognostic tools.

Yet, despite growing research efforts and many novel biomarkers showing promise, translation to clinical practice remains challenging. Often, it is our incomplete understanding of the precise mechanisms by which complement contributes to disease pathogenesis that hinders progress into novel biomarkers^2,5,22^; thus, efforts are needed to bridge the gap between basic research and clinical application.^128,129^ For example, establishing whether a finding of low levels of some serologic complement components, or high levels of urinary complement components, is due to glomerular complement activity or increased complement consumption due to overactivation remains highly context-specific and largely uncharacterized.^117,128^ Expanding our understanding of which complement components provide clinically meaningful information for specific indications will greatly facilitate the clinical application of biomarkers to support the use of novel complement inhibitors.^22,128^ Furthermore, there are important technical considerations that require uniform standardization practices. This is an important issue due to the labile nature of complement components, which makes interpretation of complement biomarkers challenging. Finally, the imminent possibility of increased FDA and European Union oversight of laboratory-developed tests for orphan diseases, such as those used to assess complement biomarkers in many CMKDs,^130,131^ could increase regulatory hurdles and result in lengthy clinical validation steps.

Importantly, as more candidate therapies move toward late-stage development, substantial opportunities exist for biomarker evaluation in controlled, larger-scale settings across varying indications. Clinical studies of complement inhibitors targeting the alternative and lectin pathways currently incorporate complement biomarker assessments and prospective studies of complement “companion” biomarkers into their protocols.^79,80,132^ In addition, the initiation and organization of an external quality assurance and standardization program for diagnostic complement laboratories (the International Union of Immunological Societies/International Complement Society Committee for the Standardization and Quality Assessment in Complement Measurements) has helped to address challenges in assay standardization and expedite progress in complement analysis, but work remains to be done.

## Supplementary Material
