# Supplementary material for: Biomarkers in the Management of Complement-Mediated Kidney Diseases in the Era of Complement Therapeutics
Source: Clin J Am Soc Nephrol. 2025 Nov 25;21(7):1245–57. doi: 10.2215/CJN.0000000967 (PMC13379139; doi:10.2215/CJN.0000000967)
Supplement: Supplementary file 1 [file cjasn-21-1245-s001.pdf]

ASN American Society of Nephrology
JASN Journal of the American Society of Nephrology
KIDNEY360 Accessing Our World From Every Angle
CJASN Clinical Journal of the American Society of Nephrology

ASN Journal Disclosure Form

As per ASN journal policy, I have disclosed any financial relationships or commitments I have held in the past 36 months as included below. I have listed my Current Employer below to indicate there is a relationship requiring disclosure. If no relationship exists, my Current Employer is not listed.

M. Chan reports the following:
Employer: University of Colorado

I understand that the information above will be published within the journal article, if accepted, and that failure to comply and/or to accurately and completely report the potential financial conflicts of interest could lead to the following: 1) Prior to publication, article rejection, or 2) Post-publication, sanctions ranging from, but not limited to, issuing a correction, reporting the inaccurate information to the authors' institution, banning authors from submitting work to ASN journals for varying lengths of time, and/or retraction of the published work.

Name: Melvin Chan
Manuscript ID: CJASN-2025-000533R1
Manuscript Title: Biomarkers in Complement-Mediated Kidney Diseases in the Era of Complement Therapeutics
Date of Completion: July 29, 2025
Disclosure Updated Date: July 29, 2025

ASN American Society of Nephrology | JASN Journal of the American Society of Nephrology | KIDNEY360 Accessing Our World From Every Angle | CJASN Clinical Journal of the American Society of Nephrology

ASN Journal Disclosure Form

As per ASN journal policy, I have disclosed any financial relationships or commitments I have held in the past 36 months as included below. I have listed my Current Employer below to indicate there is a relationship requiring disclosure. If no relationship exists, my Current Employer is not listed.

H. Cook reports the following:
Employer: Imperial College London; and Consultancy: Apellis Pharmaceuticals, Novartis, Q32 Bio, Purespring.

I understand that the information above will be published within the journal article, if accepted, and that failure to comply and/or to accurately and completely report the potential financial conflicts of interest could lead to the following: 1) Prior to publication, article rejection, or 2) Post-publication, sanctions ranging from, but not limited to, issuing a correction, reporting the inaccurate information to the authors' institution, banning authors from submitting work to ASN journals for varying lengths of time, and/or retraction of the published work.

Name: H. Terence Cook
Manuscript ID: CJASN-2025-000533R1
Manuscript Title: Biomarkers in the Management of Complement-Mediated Kidney Diseases in the Era of Complement Therapeutics,
Date of Completion: August 4, 2025
Disclosure Updated Date: April 1, 2025

ASN American Society of Nephrology | JASN Journal of the American Society of Nephrology | KIDNEY360 Accessing Our World From Every Angle | CJASN Clinical Journal of the American Society of Nephrology

ASN Journal Disclosure Form

As per ASN journal policy, I have disclosed any financial relationships or commitments I have held in the past 36 months as included below. I have listed my Current Employer below to indicate there is a relationship requiring disclosure. If no relationship exists, my Current Employer is not listed.

B. Dixon reports the following:
Employer: University of Colorado School of Medicine; Consultancy: Alexion AstraZeneca Rare Disease, Apellis Pharmaceuticals, Novartis Pharmaceuticals, Arrowhead Pharmaceuticals, Calliditas Therapeutics; Research Funding: Alexion AstraZeneca Rare Disease, Apellis Pharmaceuticals, Novartis Pharmaceuticals, Roche Genentech, Ultragenyx Pharmaceuticals; Honoraria: Alexion Pharmaceuticals; Apellis Pharmaceuticals, Novartis Pharmaceuticals, Arrowhead Pharmaceuticals; and Advisory or Leadership Role: aHUS Action Network (unpaid).

I understand that the information above will be published within the journal article, if accepted, and that failure to comply and/or to accurately and completely report the potential financial conflicts of interest could lead to the following: 1) Prior to publication, article rejection, or 2) Post-publication, sanctions ranging from, but not limited to, issuing a correction, reporting the inaccurate information to the authors' institution, banning authors from submitting work to ASN journals for varying lengths of time, and/or retraction of the published work.

Name: Bradley P. Dixon
Manuscript ID: CJASN-2025-000533R1
Manuscript Title: Biomarkers in the Management of Complement-Mediated Kidney Diseases in the Era of Complement Therapeutics
Date of Completion: July 29, 2025
Disclosure Updated Date: May 20, 2025

ASN
American Society of Nephrology
JASN
Journal of the American Society of Nephrology
KIDNEY360
Accessing Our World From Every Angle
CJASN
Clinical Journal of the American Society of Nephrology

ASN Journal Disclosure Form

As per ASN journal policy, I have disclosed any financial relationships or commitments I have held in the past 36 months as included below. I have listed my Current Employer below to indicate there is a relationship requiring disclosure. If no relationship exists, my Current Employer is not listed.

A. Frazer-Abel reports the following:
Employer: Exsera BioLab, University of Colorado School of Medicine; and Consultancy: Regeneron, Ultragenyx.

I understand that the information above will be published within the journal article, if accepted, and that failure to comply and/or to accurately and completely report the potential financial conflicts of interest could lead to the following: 1) Prior to publication, article rejection, or 2) Post-publication, sanctions ranging from, but not limited to, issuing a correction, reporting the inaccurate information to the authors' institution, banning authors from submitting work to ASN journals for varying lengths of time, and/or retraction of the published work.

Name: Ashley Frazer-Abel
Manuscript ID: CJASN-2025-000533R2
Manuscript Title: Biomarkers in the Management of Complement-Mediated Kidney Diseases in the Era of Complement Therapeutics
Date of Completion: September 23, 2025
Disclosure Updated Date: September 23, 2025

ASN American Society of Nephrology
JASN Journal of the American Society of Nephrology
KIDNEY360 Accessing Our World From Every Angle
CJASN Clinical Journal of the American Society of Nephrology

ASN Journal Disclosure Form

As per ASN journal policy, I have disclosed any financial relationships or commitments I have held in the past 36 months as included below. I have listed my Current Employer below to indicate there is a relationship requiring disclosure. If no relationship exists, my Current Employer is not listed.

D. Rizk reports the following:
Employer: University of Alabama at Birmingham; Consultancy: Novartis (Chinook) Pharmaceuticals, George Clinical, Otsuka Pharmaceuticals (Visterra), Calliditas Therapeutics (Pharmalink), LaRoche, Vera Therapeutics, BioCryst, Chugai, Biogen (HiBio), Timberlyne Therapeutics;; Ownership Interest: Reliant Glycosciences LLC; Research Funding: Travere Therapeutics (Retrophin), Calliditas Therapeutics (Pharmalink), Otsuka Pharmaceuticals (Visterra), Vertex Pharmaceuticals, Vera Therapeutics, LaRoche, Novartis Pharmaceuticals (Chinook Pharmaceuticals), Vertex, Sanofi, Dimerix;; Honoraria: Calliditas Therapeutics (Pharmalink), Novartis (Chinook) Pharmaceuticals, Otsuka Pharmaceuticals, Vera Therapeutics, BioCryst, Argenx, Alpine Immune Science, GSK;; and Advisory or Leadership Role: Novartis pharmaceuticals Steering Committee; Calliditas advisory board; Otsuka Steering Committee; Emerald (George Clinical) National Leader; Vera Therapeutics Executive Advisory Committee; Biogen Steering Committee; Roche Steering Committee.

I understand that the information above will be published within the journal article, if accepted, and that failure to comply and/or to accurately and completely report the potential financial conflicts of interest could lead to the following: 1) Prior to publication, article rejection, or 2) Post-publication, sanctions ranging from, but not limited to, issuing a correction, reporting the inaccurate information to the authors' institution, banning authors from submitting work to ASN journals for varying lengths of time, and/or retraction of the published work.

Name: Dana V. Rizk
Manuscript ID: CJASN-2025-000533R2
Manuscript Title: Biomarkers in the Management of Complement-Mediated Kidney Diseases in the Era of Complement Therapeutics
Date of Completion: September 25, 2025
Disclosure Updated Date: September 25, 2025

ASN American Society of Nephrology | JASN Journal of the American Society of Nephrology | KIDNEY360 Accessing Our World From Every Angle | CJASN Clinical Journal of the American Society of Nephrology

ASN Journal Disclosure Form

As per ASN journal policy, I have disclosed any financial relationships or commitments I have held in the past 36 months as included below. I have listed my Current Employer below to indicate there is a relationship requiring disclosure. If no relationship exists, my Current Employer is not listed.

S. Tang reports the following:
Employer: The University of Hong Kong; Consultancy: Travere Therapeutics, Boehringer Ingelheim, Novartis; Honoraria: AstraZeneca; Bayer; Baxter (Vantive); Boehringer Ingelheim; Everest Medicines, GSK, Novartis; Advisory or Leadership Role: Associate Editor, Journal of the American Society of Nephrology; and Other Interests or Relationships: Immediate Past President, Asian Pacific Society of Nephrology.

I understand that the information above will be published within the journal article, if accepted, and that failure to comply and/or to accurately and completely report the potential financial conflicts of interest could lead to the following: 1) Prior to publication, article rejection, or 2) Post-publication, sanctions ranging from, but not limited to, issuing a correction, reporting the inaccurate information to the authors' institution, banning authors from submitting work to ASN journals for varying lengths of time, and/or retraction of the published work.

Name: Sydney Tang
Manuscript ID: CJASN-2025-000533R1
Manuscript Title: Biomarkers in the Management of Complement-Mediated Kidney Diseases in the Era of Complement Therapeutics
Date of Completion: July 29, 2025
Disclosure Updated Date: May 20, 2025
